# Quality of Care for Patients Hospitalized for Heart Failure in China

**DOI:** 10.1001/jamanetworkopen.2019.18619

**Published:** 2020-01-08

**Authors:** Aakriti Gupta, Yuan Yu, Qi Tan, Shuling Liu, Frederick A. Masoudi, Xue Du, Jian Zhang, Harlan M. Krumholz, Jing Li

**Affiliations:** 1 Division of Cardiovascular Medicine, Columbia University Irving Medical Center, New York, New York; 2 Center for Outcomes Research and Evaluation, Yale School of Medicine, New Haven, Connecticut; 3 National Health Commission Key Laboratory of Clinical Research for Cardiovascular Medications, Beijing, China; 4 State Key Laboratory of Cardiovascular Disease, National Center for Cardiovascular Diseases, Beijing, China; 5 Fuwai Hospital, Chinese Academy of Medical Sciences, Beijing, China; 6 Peking Union Medical College, Beijing, China; 7 National Clinical Research Center of Cardiovascular Diseases, Beijing, China; 8 Department of Biostatistics, Yale School of Public Health, Yale University, New Haven, Connecticut; 9 Division of Cardiology, University of Colorado Anschutz Medical Campus, Aurora; 10 Heart Failure Center, Fuwai Hospital, Beijing, China; 11 Section of Cardiovascular Medicine, Yale School of Medicine, New Haven, Connecticut; 12 Department of Health Policy and Management, Yale School of Public Health, New Haven, Connecticut

## Abstract

**Importance:**

Given the emerging heart failure (HF) epidemic in China, monitoring and improving the quality of care for heart failure is a top priority.

**Objectives:**

To assess the quality of HF care provided to inpatients by examining the adherence to quality measures for HF care at the hospital level and to identify factors associated with the quality of care.

**Design, Setting, and Participants:**

In this multicenter, hospital-based, retrospective cross-sectional study in China, medical records of patients hospitalized for HF from January 1, 2015, to December 31, 2015, were analyzed from January 1, 2018, to May 20, 2019. In the first stage, simple random sampling stratified by economic-geographical regions in China was used to generate a list of participating hospitals. In the second stage, 15 538 hospitalizations from the 189 selected hospitals were systematically sampled, and 10 004 HF hospitalizations were included in the final sample.

**Main Outcomes and Measures:**

Adherence to the following 4 core performance measures at the hospital level: (1) left ventricular ejection fraction assessment during hospitalization; (2) evidence-based β-blocker (bisoprolol, carvedilol, or metoprolol succinate) for eligible patients at discharge; (3) angiotensin-converting enzyme inhibitors or angiotensin receptor blockers for eligible patients at discharge; and (4) scheduled follow-up appointment at discharge. At the hospital level, a composite performance score (ranging from 0-1) was also calculated by averaging these measures.

**Results:**

In total, 10 004 hospital admissions for HF at 189 hospitals were included in this study. The median (interquartile range [IQR]) patient age at admission was 73 (65-80) years, and 5117 (51.1%) of the patients were men. Among all hospitals, the median rate of adherence to measure 1 was 66.7% (IQR, 45.5%-80.7%; range, 0%-100%). The rate for adherence to measure 2 was 14.8% (IQR, 0%-37.5%; range, 0%-81.8%), and the rate for measure 3 was 57.1% (IQR, 36.4%-75.0%; range, 0%-100%). For measure 4, the median rate of adherence was 11.5% (IQR, 3.3%-32.8%; range, 0%-96.7%). The median (IQR) composite performance score across all hospitals was 40.0% (26.9%-51.9%), with a range from 2.2% to 85.4%. The median odds ratios of adherence were 2.2 (95% CI, 2.0-2.4) for measure 1, 2.1 (95% CI, 1.8-2.4) for measure 2, 2.4 (95% CI, 2.0-2.9 for measure 3, and 4.8 (95% CI, 3.9-5.8) for measure 4 among hospitals.

**Conclusions and Relevance:**

The findings of this study suggest that quality of care for patients with HF in China may be substandard, and there is wide heterogeneity in the quality of care for HF among hospitals. The findings also suggest the need for a national strategy to improve and standardize the quality of HF care in China.

## Key Points

**Question:**

What is the quality of care provided by hospitals in China to patients with heart failure in terms of adherence to 4 core performance measures for inpatient heart failure care?

**Findings:**

In this cross-sectional analysis of hospital records in China from 2015, which included 10 004 hospitalizations from 189 hospitals, the median composite performance score across all hospitals for the 4 core performance measures was 40.0%. The odds of receiving guideline-recommended care varied, on average, by 2- to 5-fold among hospitals for the core measures.

**Meaning:**

The findings of this study suggest that the quality of care in China for patients with heart failure may be substandard and that there may be wide heterogeneity in the quality of care for these patients among hospitals in China.

## Teaser

This cross-sectional study examines the quality of care provided by hospitals to patients with heart failure in China by assessing hospitals’ adherence to 4 core performance measures for inpatient care of heart failure.

## Introduction

Measuring and reporting on the quality of care in hospitals has been a central strategy to improve care for patients with heart failure (HF) in the United States.^1,2,3^ The US government has publicly reported on hospitals’ performance; for example, the American Heart Association’s Get With the Guidelines^4^ program provides feedback on the process and outcome measures for participating hospitals. Although such measures were instituted more than a decade ago in the United States, no similar efforts have been undertaken to examine how quality of care for HF varies at the hospital level in China, a country facing an increase in the prevalence of HF.^5^

The Chinese government has prioritized the improvement of quality of care for HF in the past decade. The Chinese Hospital Association carried out the Single Disease Quality Management Project,^6^ which included HF as one of the target diseases for quality improvement at the hospital level. In addition, HF-specific quality measures suited to the Chinese health care system were developed to lay the groundwork for future initiatives aimed at assessing and improving the quality of care.^7^ Moreover, access to inpatient health care has improved given that the State Council initiated extensive health care reform leading to attainment of universal medical insurance coverage for inpatient care.^8^ Despite these efforts, it is not known how adherence to these quality measures varies at the hospital level in China. Understanding this variation and the factors associated with higher quality of HF care may help hospitals to focus on implementing strategies for better compliance with recommended guidelines to achieve better outcomes.

Accordingly, we developed the China Patient-Centered Evaluative Assessment of Cardiac Events (PEACE 5r-HF) retrospective study of heart failure. In this study, we identified a representative sample of nonmilitary hospitals in China and a large, nationally representative sample of HF admissions in 2015. Our objective was to characterize the variation in adherence to in-hospital quality measures for HF care at the hospital level and to identify factors associated with the quality of care. These findings should provide insight into the quality of HF care in China for the first time, to our knowledge, and may help inform health care quality improvement efforts not only in China but also in other countries that share similar cultural, socioeconomic, or demographic characteristics.

## Methods

### Study Design

This study followed the Strengthening the Reporting of Observational Studies in Epidemiology (STROBE) reporting guideline. All collaborating hospitals received central ethics approval except for 15 hospitals, which obtained local approval from their internal ethics committees. According to the policies of these committees, this study was deemed to be exempt from informed consent requirements because it consisted of a review of medical records. The design of the China PEACE 5r-HF Study (investigators are listed in the eAppendix in the Supplement) has been described in detail.^9,10^

In this retrospective cross-sectional study, we used a 2-stage random sampling design to mitigate potential sampling bias and create a nationally representative sample of hospital admissions for HF in China in 2015 (eFigure in the Supplement). All analyses for this study were performed from January 1, 2018, to May 20, 2019. In the first stage, we identified hospitals in China providing care for acute HF. We excluded military hospitals, prison hospitals, and those providing only traditional Chinese medicine–related services. We stratified eligible hospitals into 5 economic and geographical regions (Eastern rural, Central rural, Western rural, Eastern urban, and Central-Western urban). We randomly sampled tertiary and secondary hospitals in the 2 urban regions and all central hospitals in the 3 rural regions. In the present study, admissions to nonteaching hospitals, nontertiary care hospitals, and central hospitals in the rural regions constituted 37.4%, 58.2%, and 56.4%, respectively, of the participating population.

In the second stage, we used systematic random sampling procedures to select patients hospitalized for HF from the local hospital database of each sampled hospital. We systematically sampled 15 538 hospitalizations from the 189 selected hospitals. We identified HF cases based on the *International Statistical Classification of Diseases, Tenth Revision, Clinical Modification*^11^ *(ICD-10-CM)* codes (I50.xx, I11.0x, I13.0x, or I13.2x) when available or through principal diagnosis terms stated in the discharge notes. The mean (range) proportion of cases sampled at a hospital was 9.1% (5.2%-100%). Central data abstraction with use of standardized definitions was performed, and 2 contracted vendors abstracted details of each patient’s hospitalization using their medical records.^9^ After we sampled cases at each hospital, we assigned a unique study identification number to each case. Site investigators scanned complete hospital medical records of all sampled patients, with direct identifiers concealed (name, national identification number, and contact information). All parts of the medical record were required for scanning. The data were evaluated to ensure completeness, quality, and concealment of direct identifiers. Incomplete or poorly scanned records were scanned again. One of us (J.L.) and other research staff from the coordinating center visited 20 sites to assist in processing the sampled cases. We monitored each stage, including case searching, sampling, record scanning, and data abstraction and cleaning, to ensure data quality. To ensure the accuracy of medical records abstraction exceeding 98%, we used double entry for sections that did not need interpretation, such as diagnostic procedure reports, laboratory tests, and physician orders, and double auditing for sections that needed interpretation, such as admission notes, progress notes, procedure notes, and discharge summaries.

### Participants

The study included patients aged 18 years or older, hospitalized from January 1, 2015, to December 31, 2015, with a principal discharge diagnosis of HF. Patients with an admission diagnosis of acute myocardial infarction were excluded because their primary reason for admission was not HF. Patients who were classified as having New York Heart Association class I HF were also excluded owing to the higher specificity of the diagnosis of acute decompensated HF. We observed that patients with chronic HF who were hospitalized could have HF documented as one of their discharge diagnoses even if acute decompensated HF was not the primary reason for their admission. Consequently, for further specificity, we required that patients presented with typical symptoms or signs of acute HF and that they received typical HF treatments, including diuretics or inotropes, during hospitalization. Typical symptoms included orthopnea, paroxysmal nocturnal dyspnea, dyspnea at rest, dyspnea on exertion, and edema and/or oliguria. Typical signs included jugular venous distension, hepatojugular reflux, pulmonary rales, S3 heart sound, and lower-extremity edema. Thus, 10 004 of the 15 538 HF hospitalizations originally sampled were included in the final sample.

### Data Collection

The data were collected from July 20, 2016, to January 1, 2018. We abstracted data for demographic characteristics, clinical characteristics, diagnostic tests, and treatments from medical records. Information about the medical record abstraction has been described in detail.^9^ We collected all documented left ventricular ejection fraction (LVEF) assessments during hospitalization and no longer than 1 month before admission. Medical history and comorbidities were obtained from the documented history in the admission notes, discharge diagnosis, or positive laboratory test results.

We also used a survey to collect data on hospital characteristics, including teaching status, medical university affiliation, number of beds, and whether the hospital had a catheterization laboratory, an independent department of cardiology, cardiovascular intensive care unit, a cardiologist in the emergency department, and the capability to perform coronary artery bypass graft (CABG). Hospitals were classified according to their government-defined level in 2015. Secondary hospitals have at least 100 inpatient beds and the capacity to treat local populations of at least 100 000 patients, whereas tertiary hospitals are larger centers that provide more advanced care.

### Performance Measures

We used 4 performance measures adapted from the 2014 guidelines for HF management in China,^12^ the 2013 American Heart Association/American College of Cardiology guidelines,^13^ and the Get With the Guidelines program^4^ for the management of HF: (1) LVEF assessment during hospitalization; (2) evidence-based β-blocker (bisoprolol, carvedilol, or metoprolol succinate) for HF with reduced ejection fraction (HFrEF) (LVEF <40%) at discharge; (3) angiotensin-converting enzyme inhibitors (ACEIs) or angiotensin receptor blockers (ARBs) for HFrEF at discharge; and (4) scheduled follow-up appointment at discharge.

For the evaluation of LVEF assessment during hospitalization, patients with a length of stay of less than 24 hours were excluded owing to the possibility that there was insufficient time for them to undergo an echocardiogram. For β-blockers and ACEIs or ARBs for HFrEF at discharge, patients who died or withdrew care, did not receive LVEF assessment or had a documented LVEF of 40% or more, or had known contraindications to therapy were excluded (eTable 1 in the Supplement). For scheduling a follow-up appointment at discharge, patients who died or withdrew care during hospitalization were excluded.

### Statistical Analysis

The data obtained from the hospital records were analyzed from January 1, 2018, to May 20, 2019. Adherence to each of the 4 performance measures at the hospital level was calculated for each hospital by determining the proportion of indicated patients who had each measure assessed. Hospitals with fewer than 25 total admissions or 5 eligible patients for a given measure were excluded. A composite performance score was calculated depending on the number of the performance measures attained divided by the number potentially indicated for each patient. The score was averaged at the hospital level for all patients to generate a composite performance score (continuous score ranging from 0-1).

To describe the variation among hospitals, we computed the median odds ratio (OR) for performance measures. When applied to multilevel data, such as information about patients treated at different hospitals, the median OR quantifies the mean difference in treatment rates across hospitals. The interpretation of the median OR is the mean difference of a statistically identical patient being treated at a given hospital compared with another random hospital. As a result, a high median OR indicates substantial between-hospital variation in the relevant treatment rate, whereas a low median OR suggests that hospital performance was similar across institutions. To calculate the median OR, we used hierarchical logistic regression to model the 4 performance measures and the composite score as a function of patient characteristics as well as a hospital-level random effect to measure between-hospital variance. We used the intraclass coefficient (ICC) to estimate the proportion of variance in performance that was attributable to between-hospital variations. We calculated the ICC from these hierarchical logistic models using the following equation: ICC = SE^2^ / (SE^2^ + (π^2^) / 3), where SE is the standard error of the random hospital intercept.^14^

To examine associations between hospital characteristics and the composite performance score, we divided hospitals into quartiles based on their respective composite performance score (with the bottom quartile containing hospitals with the lowest composite scores and the top quartile containing hospitals with the highest composite scores). Within each performance score quartile, a proportion was calculated for each categorical hospital characteristic and a median was calculated for each continuous hospital characteristic. To examine whether hospital characteristics differed across quartiles, continuous and categorical covariates at the hospital level were tested for trends using linear regression with the median composite score as a continuous outcome variable. To further assess which hospital characteristics were associated with a better performance score, multivariable linear regression modeling with the median composite performance score as the continuous outcome variable was performed. We reported percentage change in the composite performance score with 95% CIs.

For the 4 performance measures, the data were complete for all participants. The variable with the highest proportion of missing values was glomerular filtration rate at 5.8%. For other variables, data were missing from less than 1% of the participants, and we imputed the median values for these variables for participants for whom these data were missing. All comparisons were 2-tailed, with *P* < .05 considered statistically significant. Statistical analysis was performed with SAS software, version 9.3 (SAS Institute Inc).

## Results

### Baseline Characteristics

In total, 10 004 hospital admissions for HF at 189 hospitals were included in this study. The median (interquartile range [IQR]) patient age at admission was 73 (65-80) years, and 5117 (51.1%) of the patients were men (eTable 2 in the Supplement). Details on the characteristics of this patient population were published previously.^15^ Of the 6258 patients for whom LVEF was assessed, 1374 (22.0%) had an LVEF less than 40%. Angiotensin-converting enzyme inhibitors or ARBs were prescribed at discharge to 648 of 1266 eligible patients (51.2%) with left ventricular systolic dysfunction (LVSD), and evidence-based β-blockers were prescribed at discharge to 262 of 1327 eligible patients (19.7%) with LVSD. A follow-up appointment was scheduled for 2325 of 9662 eligible admissions (24.1%) (eTable 3 in the Supplement).

### Compliance With Performance Measures at the Hospital Level

Across all hospitals, the median rate of performance of LVEF assessment in patients hospitalized for HF was 66.7% (IQR, 45.5%-80.7%; range, 0%-100%), prescription of guideline-recommended β-blockers was 14.8% (IQR, 0%-37.5%; range, 0%-81.8%), prescription of ACEIs or ARBs in eligible patients was 57.1% (IQR, 36.4%-75.0%; range, 0%-100%), and scheduling follow-up appointments at discharge was 11.5% (IQR, 3.3%-32.8%; range, 0%-96.7%) (Figure and Table 1). The median composite performance score across all hospitals included in our study was 40.0% (IQR, 26.9%-51.9%; range, 2.2%-85.4%).

**Figure.**
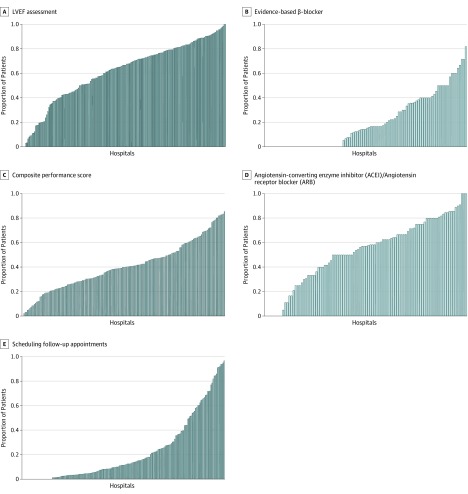
Hospital-Level Variation in Adherence to Core Performance Measures for Inpatient Heart Failure Care and Their Composite Score Each bar represents a hospital. The scale on the y-axis represents the proportion of patients who received a particular measure. LVEF indicates left ventricular ejection fraction.

**Table 1.** Hospital-Level Variation in Adherence to Core Performance Measures for Inpatient Heart Failure Care

Core Performance Measure	Median (IQR), %	Median Odds Ratio (95% CI)	ICC
LVEF assessment during hospitalization	66.7 (45.5-80.7)	2.2 (2.0-2.4)	0.17
ACEIs or ARBs for HFrEF at discharge	57.1 (36.4-75.0)	2.4 (2.0-2.9)	0.21
Evidence-based β-blocker for HFrEF at discharge	14.8 (0-37.5)	2.1 (1.8-2.4)	0.16
Scheduled appointment at discharge	11.5 (3.3-32.8)	4.8 (3.9-5.8)	0.46

Abbreviations: ACEI, angiotensin-converting enzyme inhibitor; ARB, angiotensin receptor blocker; HFrEF, heart failure with reduced ejection fraction; ICC, intraclass coefficient; IQR, interquartile range; LVEF, left ventricular ejection fraction.

There was statistically significant variability in adherence to all 4 performance measures associated with differences among hospitals (Table 1). Of the total variability in assessment of LVEF, approximately 17% was associated with differences among hospitals (ICC, 0.17). Similarly, significant variability in prescription of ACEIs or ARBs (ICC, 0.21), prescription of β-blockers (ICC, 0.16), and scheduling follow-up appointments (ICC, 0.46) was associated with differences among hospitals. The median OR for LVEF assessment during hospitalization was 2.2 (95% CI, 2.0-2.4), suggesting that the mean odds of a patient receiving an LVEF assessment during hospitalization would differ 2.2 times from 1 randomly selected hospital to another hospital. Similarly, the median ORs for prescription of guideline-recommended β-blockers, prescription of ACEIs or ARBs, and scheduling a follow-up appointment after discharge were 2.1 (95% CI, 1.8-2.4), 2.4 (95% CI, 2.0-2.9), and 4.8 (95% CI, 3.9-5.8), respectively.

### Hospital-Level Factors Associated With Composite Performance Score

In stratified analyses of the composite performance score, hospitals in the top quartile of composite performance had a higher proportion of tertiary hospitals (68.2% vs 11.6%; *P* < .001), urban hospitals (61.4% vs. 22.7%; *P* < .001), teaching hospitals 81.4% vs. 36.4%; *P* < .001), and medical university–affiliated hospitals (59.1% vs. 15.9; *P* < .001) and were more likely to be located in the Eastern region (65.9% vs. 29.6%; *P* < .001) than those in the bottom quartile (Table 2). Additionally, hospitals in the top quartile of performance had a higher median number of beds (1094; IQR, 549-1780 vs 260; IQR 150-450) (*P* < .001) and were more likely to have cardiac catheterization laboratories (83.3% vs. 25%; *P* < .001), CABG capability (46.5% vs. 2.3%; *P* < .001), an independent department of cardiology (100% vs. 45.4%; P < .001), and a cardiac care unit (69% vs. 36.4%, *P* < .001) than hospitals in the bottom quartile.

**Table 2.** Hospital Characteristics Across Quartiles of Composite Performance Score for Heart Failure Care

Characteristic	Overall (n = 176)	Q1 (n = 44)	Q2 (n = 44)	Q3 (n = 44)	Q4 (n = 44)	*P* Value
Composite performance score, median (range), %	40.0 (2.2-85.4)	19.3 (2.2-26.9)	32.6 (26.9-40.0)	45.5 (40.0-51.9)	65.1 (51.9-85.4)	NA
Tertiary hospital, No. (%)	75 (42.6)	5 (11.6)	17 (37.8)	23 (53.5)	30 (68.2)	<.001
Location, No. (%)						
Eastern	72 (40.9)	13 (29.6)	14 (31.1)	16 (37.2)	29 (65.9)	<.001
Central	47 (26.7)	15 (34.1)	15 (33.3)	11 (25.6)	6 (13.6)	.02
Urban, No. (%)	78 (44.3)	11 (22.7)	17 (37.8)	24 (55.8)	27 (61.4)	<.001
Teaching hospital, No. (%)	110 (62.9)	16 (36.4)	28 (62.2)	31 (72.1)	35 (81.4)	<.001
Medical university–affiliated hospital, No. (%)	54 (30.7)	7 (15.9)	6 (13.3)	15 (34.9)	26 (59.1)	<.001
Catheterization laboratory, No. (%)	104 (59.8)	11 (25.0)	26 (57.8)	32 (74.4)	35 (83.3)	<.001
Beds, median (IQR), No.	637 (280-1100)	260 (150-450)	608 (260-1000)	775 (540-1300)	1094 (549-1780)	<.001
CABG capability, No. (%)	40 (23.0)	1 (2.3)	5 (11.1)	14 (32.6)	20 (46.5)	<.001
Independent department of cardiology, No. (%)	140 (79.6)	20 (45.4)	36 (80.0)	40 (93.0)	44 (100)	<.001
Cardiovascular care unit, No. (%)	99 (56.9)	16 (36.4)	26 (57.8)	28 (65.1)	29 (69.0)	<.001
Cardiologist in emergency department, No. (%)	77 (44.8)	13 (30.2)	24 (54.6)	21 (48.8)	19 (45.2)	.25

Abbreviations: CABG, coronary artery bypass graft; IQR, interquartile range; NA, not applicable; Q, quartile.

We found that several hospital characteristics were significantly associated with a higher composite performance score according to stepwise linear regression (Table 3). Eastern hospitals had a mean composite performance score that was 7.6% (95% CI, 2.5%-12.6%; *P* = .003) higher than that of hospitals in other regions. Teaching hospitals and CABG capability were associated with an increase of 5.6% (95% CI, 0.1%-11.2%; *P* = .047) and 8.3% (95% CI, 2.1%-14.5%; *P* = .009), respectively, in the composite performance score. An independent cardiology department was associated with an increase of 16.5% (95% CI, 9.9%-23.1%; *P* < .001) in the performance score.

**Table 3.** Hospital Characteristics Associated With Better Composite Performance Score in Multivariable Linear Regression

Variable	% Change in Composite Performance Score (95% CI)	*P* Value
Eastern region	7.6 (2.5-12.6)	.003
Teaching hospital	5.6 (0.1-11.2)	.05
CABG capability	8.3 (2.1-14.5)	.009
Independent department of cardiology	16.5 (9.9-23.1)	<.001

Abbreviation: CABG, coronary artery bypass graft.

## Discussion

This is the first study, to our knowledge, to evaluate variation in HF care among hospitals in China. We found pervasive gaps in the quality of care with respect to adherence to all 4 core performance measures: LVEF assessment during hospitalization, guideline-recommended β-blockers (bisoprolol, carvedilol, or metoprolol succinate) for HFrEF at discharge, ACEI or ARB therapy for HFrEF at discharge, and scheduling a follow-up appointment at discharge. We also found substantial heterogeneity in the quality of care among the worst- and best-performing hospitals. The mean odds of receiving guideline-recommended care varied 2.1-fold to 4.8-fold between hospitals for the core measures. Moreover, a considerable proportion of this variation was attributable to between-hospital differences in care provision. Hospitals with CABG capability and those located in the Eastern region or that had an independent department of cardiology were more likely to perform better.

The present study contributes to scientific literature in several ways, although the findings of poor adherence to guideline-recommended care for HF are consistent with those of previous studies conducted in China.^16,17,18,19^ First, the size and scope of the present study, one of the largest on HF in China, allowed us to draw national-level inferences about HF care at the hospital level. Our findings revealed a poor median rate of adherence to all 4 core measures ranging from 11.5% for scheduling a follow-up appointment to 66.7% for LVEF assessment. Moreover, the adherence rates remained substandard across urban or rural care settings. These rates are poorer compared with similar analyses from the United States^20^ and Europe,^21^ where hospital-level adherence rates to these measures have been more than 70%. Low adherence rates for LVEF assessment among hospitals reflect a substantial opportunity for targeting subsequent quality improvement initiatives. Not only could the initiatives to improve LVEF assessment improve performance on this particular measure, but it could also enable identification of more patients eligible for quality measures applicable to those with HF and an LVEF less than 40% (prescription of evidence-based β-blockers and ACEI or ARBs at discharge) and improve care for these patients.

We provide new information about hospital-level characteristics associated with adherence to core measures for HF care. Hospitals with independent cardiology departments and CABG capability and those located in the Eastern region of China had a better performance compared with hospitals without those resources that were located in other regions, signifying the importance of superior access to knowledge and health care resources. Prior studies from other countries have noted differences in the quality of HF care based on the academic status,^22^ urban location,^23^ and larger size of hospitals.^24,25^ The association between the presence of an independent cardiology department and the composite performance score was strong (16.5%) and likely owing to better knowledge and implementation of current HF guidelines by virtue of having dedicated cardiology departments. Of note, better-performing hospitals were more frequently located in urban areas and in the Eastern region. Differences in the insurance and the extent of coverage may partially account for these differences. China’s social health insurance, including the rural new cooperative medical scheme (NCMS, launched in 2003), urban resident-based basic medical insurance (URBMI, launched in 2007), and urban employee-based basic medical insurance (UEBMI, launched in 1998), have rapidly expanded during the past decade and at present cover almost the entire population in China. Although China has achieved universal health coverage in recent years, the quality and extent of care and coverage may vary widely. Based on data from the National Health Service survey,^26^ the reimbursement rates for hospital admission were 68.8% for patients with UEBMI, 53.6% for those with URBMI, and 50.1% for those with rural NCMS in 2013. Eastern China has the highest rates of URBMI and UEBMI coverage, and Western and Central China have a higher rate of NCMS coverage. Rural populations have more restricted access to health care services than do urban residents and have a larger financial burden, mainly owing to low funds for NCMS. Efforts to consolidate these fragmented social health insurance plans may help to reduce these disparities.

This is the first report, to our knowledge, highlighting considerable heterogeneity in HF care among individual hospitals in China. Quality differences among the hospitals with the best and worst performance were distinct. We found that adherence to the 4 quality measures ranged from 0% in poor-performing hospitals to 100% in the best-performing hospitals. Although there was a 2 to 3 times difference in the odds of receiving LVEF assessment or prescription of ACEIs or ARBs or of β-blockers between 2 randomly selected hospitals, this variation was even greater (approximately 5 times greater) for scheduling a follow-up appointment. Adherence to this measure is likely highly variable given that it is resource intensive to schedule a follow-up appointment, and the evidence supporting it is less strong than pharmacological interventions.^13,27,28^ Similarly, greater heterogeneity for this measure has been observed in a hospital-level analysis from the National Heart Failure Audit in the United Kingdom.^21^ A hospital-level analysis for quality of care for ST-segment elevation myocardial infarction in China also revealed substantial variation among hospitals, even though the absolute adherence to quality measures was much higher than that observed for HF in the present study.^29^ Our findings may have implications for policies targeting organizational changes in individual hospitals to reduce the gaps in care for patients with HF.

Experience from the United States provides lessons for China. Studies conducted in the United States also reported marked gaps in HF care with substantial variation across institutions reported approximately 2 decades ago.^30,31^ In response, several national initiatives focused on strategies to improve adherence and publicly reported process quality measures.^23,32^ Not only did the median performance across hospitals improve, but the performance gap decreased among hospitals by 2011.^4^ The Chinese government has established a panel of HF-specific quality measures to characterize hospital performance.^6^ Although quality measurement may stimulate some improvement, it will likely not suffice by itself. Efforts such as the launch of national hospital quality monitoring systems, concomitant public reporting, policies in favor of top-performing hospitals, and quality improvement programs tailored for poor-performing hospitals are warranted.

### Limitations

This study has limitations. First, we relied on information documented in medical records, and there may be hospital- or clinician-level variation in the quality or completeness of documentation. Second, LVEF was not assessed in approximately one-third of the patients. Therefore, we could not accurately estimate the use of guideline-recommended therapies. However, this fact is a noteworthy finding given that evaluation of LVEF in patients admitted with acute HF is a core performance measure. In addition, although our study was nationally representative and the results demonstrate the range of performance achieved by about 189 Chinese hospitals that provide inpatient care for HF, we focused on a single condition; it is unknown whether our findings could be extrapolated to other conditions.

## Conclusions

The quality of HF care may be substandard at the hospital level in China. Despite clear guidelines from several international societies, adherence to core measures for HF appears to be highly variable across hospitals in China, suggesting that patients with acute HF may be treated differently depending on where they are admitted. Our findings support broad-based opportunities to improve HF care in China and suggest the need for a national strategy to reduce heterogeneity in the quality of care for HF.

## Notes

Supplement. **eAppendix.** China PEACE 5r-HF Study Site Investigators by Hospital **eTable 1.** Definition for Indications and Contraindications for Medications **eTable 2.** Baseline Characteristics of Patients Hospitalized With HF in China **eTable 3.** Patient-Level Adherence to Core Performance Measures for Inpatient Heart Failure Care **eFigure.** Flowchart of Sampling, Data Collection, Cleaning, and Analysis
