# Supplementary material for: Quality of Care for Patients Hospitalized for Heart Failure in China
Source: JAMA Netw Open. 2020 Jan 8;3(1):e1918619. doi: 10.1001/jamanetworkopen.2019.18619 (PMC6991250; doi:10.1001/jamanetworkopen.2019.18619)
Supplement: Supplement. — eAppendix. China PEACE 5r-HF Study Site Investigators by Hospital eTable 1. Definition for Indications and Contraindications for Medications eTable 2. Baseline Characteristics of Patients Hospitalized With HF in China eTable 3. Patient-Level Adherence to Core Performance Measures for Inpatient Heart Failure Care eFigure. Flowchart of Sampling, Data Collection, Cleaning, and Analysis [file jamanetwopen-3-e1918619-s001.pdf]

Supplementary Online Content

Gupta A, Yu Y, Tan Q, et al. Quality of care for patients hospitalized for heart failure in China . *JAMA Netw Open.* 2020;3(1):e1918619. doi:10.1001/jamanetworkopen.2020.18619

eAppendix. China PEACE 5r-HF Study Site Investigators by Hospital
eTable 1. Definition for Indications and Contraindications for Medications
eTable 2. Baseline Characteristics of Patients Hospitalized With HF in China
eTable 3. Patient-Level Adherence to Core Performance Measures for Inpatient Heart Failure Care
eFigure. Flowchart of Sampling, Data Collection, Cleaning, and Analysis

This supplementary material has been provided by the authors to give readers additional information about their work.

© 2020 Gupta A et al. *JAMA Network Open.*

eAppendix: China PEACE 5r-HF Study Site Investigators by Hospital

Kaifeng Integrative Medicine Hospital, Qin Lei, Liu Jieyun; Haiyan County People's Hospital, Xiao Chunhui, Lu Zhihua; Panyu District, Guangzhou City Central Hospital, Guoqin Chen; Harbin Daoli District People's Hospital, Yongfan Jin; Second Affiliated Hospital of Hebei North University, Wenhui Li; Hohhot, Hohhot Saihan District Second People's Hospital, Rongjuan Zhang; Huzhou Nanxun People's Hospital, Fuqin Zhu; Huangshan Third People's Hospital, Changjie Hong; Jining City People's Hospital, Chuanxin Li; Jiaxing Nanhu District Central Hospital east, Zhihua Sun; Laibin Xingbin Bayi Hospital, Chunhua He; Hongzhou District, Lanzhou City People's Hospital, Ping Zhang; Fuzhou, Jiangxi Province Linchuan People's Hospital, Youzhi Zhan; Qujing Qilin District People's Hospital, Fuyong Li; Qinghai Province Fifth People's Hospital, Hong Wu; Qujiang District People's Hospital, Jianfan Chen; Yuhong District, Shenyang City People's Hospital, Meijuan Piao; Taiyuan Xinghua Ridge Central Hospital, Yue Qu; Kaiping Hospital in Tangshan City, Yanmin Yao; Caidian District, Wuhan City, Hubei Province People's Hospital, Baojun Hou; Xining Third People's Hospital, Qing Feng; Yangling Demonstration Area Hospital, Xiaoqiang Yang; Yichun Second People's Hospital, Ying Yuan; Zhengzhou People's Hospital, Hengliang Liu; Chongqing Sixth People's Hospital, Yonghong Huang; Nanchuan District People's Hospital of Chongqing, Lingxian Zeng; Aba Tibetan and Qiang Autonomous Prefecture People's Hospital, Bo Cai; Alxa League Hospital, Shiguo Hao; Anshan City Double Hill Hospital, Rui Xiao; Anshan Mayor Hospital, Xiang Jin; Baiquan County People's Hospital, Yachen Zhang; Baotou Fourth Hospital, Baohong Zhang; Baoding Second Central Hospital, Guang Ma; Beihua University Hospital, Feng Sun; Peking University People's Hospital, Hong Chen; Peking University Shenzhen Hospital, Chun Wu; Beijing Watson Hospital, Lihua Shang; Beipiao Central Hospital, Han Yu; Bortala Mongol Autonomous Prefecture People's Hospital, Ping Chen; Changtu County First Hospital, Mingbao Sun; Chongren County People's Hospital, Chun Yuan; Zhongshan Hospital Affiliated to Dalian University, Qin Yu; Dalian Central Hospital, Yongchao Zhi; Dashiqiao Central Hospital, Juan Huang; Daofu County People's Hospital in Sichuan Province, Jiekang Liu; Dingyuan County General Hospital, Xinming Ma; Dongyang City People's Hospital, Liang Lu; Dunhua City Hospital, Fanju Meng; Fenghuang County, Hunan Province People's Hospital, Guangyong Liu; Fengshan County People's Hospital, Wen Long; Fujian Provincial Hospital, Yansong Guo; Fuzhou First Affiliated Hospital of Fujian Medical University, Yan Zhang; Fugu County People's Hospital, Ruijun Hao; Gannan County People's Hospital, Mei Chen; Gongcheng Yao Autonomous County People's Hospital, Mingfang Feng; Gongshan Dulong Nu Autonomous County People's Hospital, Xiaoping Wu; Guyuan Yuanzhou District People's Hospital, Xiaoping Gao; Guangchang County People's Hospital, Xiang Fu; Guiping City People's Hospital, Guang Chen; Guangyuan First People's Hospital, Tianxun Wang; Guiyang Medical College Hospital, Lirong Wu; Guilin People's Hospital, Diguang Pan; Harbin two four two hospitals, Jiubin Sun; Second Affiliated Hospital of Harbin Medical University, Bo Yu; Haimen People's Hospital, Jie Wu; Hainan West Central Hospital (Pizhou First People's Hospital), Zhongwei Wu; Hainan Medical College Hospital, Yueqiong Kong; Hellinger County People's Hospital, Yongshuan Wu; Helong City People's Hospital, Yinglin Cui; First Affiliated Hospital of Hebei North University, Fangjiang Li; First Affiliated Hospital of Henan University of Science and Technology, Pingshuan Dong; Henan Provincial People's Hospital, Chuanyu Gao; Heze City Hospital, Wentang Niu; Hegang Mining Group Co., Ltd. General Hospital, Xiaowen Pan; Chenxi County People's Hospital, Xuejin He; County People's Hospital, Shengcheng Zhou; Jianghua Yao Autonomous County People's Hospital, Rongjun Wan; Hunan Provincial People's Hospital Mawangdui Hospital, Zhiyi Rong; Hunan Provincial People's Hospital, Xing Wang; Xupu County People's Hospital, Yangzhou Liang; Yongzhou City, Hunan Province Central Hospital, Bin Liu; Yuanling County People's Hospital, Rong Cai; Huayin City People's Hospital, Aiping Wang; Guang'an Huayang City People's Hospital of Sichuan Province, Zhihong Zhang; Hunchun City Hospital, Lijun Yu; Huizhou City People's Hospital, Yuansheng Shen; Jixi City People's Hospital, Jia Wang; Jize County Hospital, Qiu'e Guo; Ji'an City, Jiangxi Province

© 2020 Gupta A et al. *JAMA Network Open.*

People's Hospital, Xueqiao Wang; Jilin Province, Jilin Integrative Medicine Hospital, Jianping Shi; Jingyu County People's Hospital, Yuhui Lin; Jilin Provincial People's Hospital, Yuming Du; First Affiliated Hospital of Jiamusi University, Zhaofa He; Jiangxi Provincial People's Hospital, Qing Huang; Jinning County People's Hospital, Lihua Gu; Jingzhou Central Hospital, Jin Xie; Jingxing Hospital, Zhenhai Zhao; Jingxi County People's Hospital, Wen Liang; Jiuquan City People's Hospital, Yaofeng Yuan; Kangbao County People's Hospital, Ruiqing Zhao; Keshiketeng Banner Hospital, Li Wang; Lanping Bai Minority Autonomous County People's Hospital, Runxiang He; Yueqing People's Hospital, Xudong Yu; Laoting County Hospital, keyong Shang; Liaoyang City Central Hospital, Yingying Li; Liaoyuan Second People's Hospital, Aimin Zhang; Liaoyuan City Central Hospital, Fenghua Wu; Lindian County Hospital, Wenzhou Li; Linxiang City People's Hospital, Xiyuan Zhao; Liujiang County People's Hospital, Meifa Wei; Shougang Shuicheng Iron and Steel (Group) Co., Ltd. General Hospital, Min Zhang; Longyan City, Fujian Province First Hospital, Haiming Yi; Luxi County People's Hospital of Jiangxi Province, Feilong Duan; Luchuan County People's Hospital, Min Feng; Luyi County People's Hospital, Yuanxun Xu; Shijiazhuang City Luan City People's Hospital, Ruigang Zhao; Macheng City People's Hospital, Hongzhuan Cai; Mengcheng County First People's Hospital, Gaofeng Guo; Menglian Dai Lahu Wa Autonomous County People's Hospital, Xiang Li; Biyang County People's Hospital, Weijuan Zhou; Minxian County People's Hospital in Gansu Province, Yuhong Liu; Muli Tibetan Autonomous County People's Hospital, Hui Peng; Nanan Hospital, Duanping Dai; Nanjing First Hospital, Shaoliang Chen; Nantong City Maternal and Child Health Hospital, Song Chen; Nanyang Central Hospital, Shouzhong Yang; Ningwu County People's Hospital, Junhu An; Piao County People's Hospital of Shanxi Province, Jinsong Jiao; Puding County People's Hospital, Wei Jiang; Qinshui County People's Hospital, Hehua Zhang; Qinyang City People's Hospital, Xiaowen Ma; Qinghai Red Cross Hospital, Yanmei Shen; Quzhou City People's Hospital, Xiaoming Tu; Queshan County People's Hospital, Guoyin Fan; Rongjiang County People's Hospital, Fangning Wang; Rudong County People's Hospital, Dongmei Liu; Ruyang County People's Hospital, Chengning Shen; Heze City, Shandong Province Chengwu County People's Hospital, Fengqin Liu; Lucheng People's Hospital of Shanxi Province, Yunke Zhou; Shangluo City Central Hospital, Yazi Yu; Shangqiu Fourth People's Hospital, Jianjun Pan; Shangqiu Long March People's Hospital, Qian Wang; Shanghai Jiao Tong University School of Medicine Ruijin Hospital, Xiaoxiang Yan; Shaoyang County People's Hospital, Kaiyou Wu; Shengsi People's Hospital, Songguo Wang; Huairen County People's Hospital, Ling Tong; Ying County People's Hospital, Wenbing Zhao; Inner Mongolia Siziwangqi People's Hospital, Hongtu Zhang; Sunan Yugur Autonomous County People's Hospital, Zhansheng Ba; Tianjin Jinghai County Hospital, Yuling Zhang; Tianjin Medical University General Hospital, Yuemin Sun; Tongliao City Horqin District First People's Hospital, Junping Fang; Tongchuan Mines Central Hospital, Guojiong Jia; Tongliang County People's Hospital, Guofu Li; Wencheng County People's Hospital of Zhejiang Province, Junlu Wang; Wuhai People's Hospital, Zhaohai Zhou; Wulateqianqi People's Hospital, Jinlan Xu; Wulanchabu City Central Hospital, Dajun Liu; Wuchuan City People's Hospital, Yuanming Yi; Wuqiang County People's Hospital, Binglu Liu; Wuyishan City, Fujian Province Hospital, Qingfei Lin; Xi'an First Hospital, Yuqiang Ji; Xiangtan County People's Hospital, Xiaoshan Yang; Gyantse People's Hospital, Ouzhu Danzeng; Xinmi City First People's Hospital, Jie Dou; Xinshao County People's Hospital, Jintang Wang; Xinghai County People's Hospital, Guohui Zhou; Xingshan County People's Hospital in Hubei Province, Shubing Wu; Xing County, Shanxi Province People's Hospital, Aiping Lv; Xiuwu County People's Hospital, Jianbao Chang; Xuanhan County People's Hospital, Xuan Ma; Yanqing District Hospital in Beijing, Li Yang; Yanggao County People's Hospital, Zhiru Peng; Yitong County People's Hospital of Jilin Province, Haifeng Wang; Yongxing County People's Hospital, Shengyong Deng; Yuyao City, Zhejiang Province People's Hospital, Lian Chen; Yunlong County People's Hospital, Jianxun Yang; Yuncheng City, Shanxi Province Central Hospital, bo Wang; Zaduo County People's Hospital of Qinghai Province, Cairen Nima; Zhangjiachuan Hui Autonomous County People's Hospital, Shitang Gao; Changjiang Shipping

© 2020 Gupta A et al. *JAMA Network Open.*

General Hospital, Xiuqi Li; Taizhou Hospital in Zhejiang Province, Yafei Mi; Zhijiang City People's Hospital, Bing Zhang; Fuling Central Hospital in Chongqing, liquan Xiang; Zhouning County Hospital, Banghua He; Zhuoni County People's Hospital, Hong Li; Zhuozi County People's Hospital, julong Hao; Fourth People's Hospital of Zigong City, Yong Yi; Zuo Yun County People's Hospital of Shanxi Province, Ru Duan

© 2020 Gupta A et al. *JAMA Network Open.*

eTable 1. Definition for Indications and Contraindications for Medications

Eligible patients were defined as patients with indication and without contraindications for following medications.

Medications	Indication	Contraindications
Angiotensin-converting-enzyme inhibitors (ACEI) or angiotensin receptor blockers (ARB)	left ventricular ejection fraction (LVEF) < 40%	Allergy to ACEI/ARB<br>Hyperkalemia (serum potassium >5.5 mmol/L)<br>Creatinine >2.5 mg/dL in men or >2.0 mg/dL in women<br>Systolic blood pressure <90mmHg<br>Pregnancy<br>Moderate-to-severe aortic stenosis<br>Other documented contraindications
β blockers	LVEF < 40%	Allergy to β blockers<br>Asthma<br>Second or third degree atrioventricular block with no pacemaker implanted<br>Systolic blood pressure <90mmHg<br>Bradycardia (heart rate <55 beats/min) without taking a β blocker<br>Other documented contraindications

Number of patients having each contraindications

	**Overall** (n=10004)	%
**Contraindications for ACEI/ARB**	881	8.81
Allergy	0	0
Hyperkalemia	133	1.33
Hypotension	135	1.35
Moderate-severe aortic stenosis	72	0.72
Pregnancy	11	0.11
Renal dysfunction	621	6.21
**Contraindications for β blocker**	355	3.55
Allergy	3	0.03
Asthma	94	0.94
Bradycardia on day of discharge or day prior to discharge while not on a β blocker	146	1.46
Hypotension	135	1.35
Second- or third-degree heart block and does not have a pacemaker	0	0

ACEI, angiotensin-converting enzyme inhibitor; ARB, angiotensin receptor blocker

© 2020 Gupta A et al. *JAMA Network Open.*

eTable 2. Baseline Characteristics of Patients Hospitalized With HF in China

	**Overall** (n=10004)
**Social demographic**	
Age (years), median, IQR	73 (65, 80)
Female (%)	48.9
Han (%)	90.6
Past or current smoking (%)	21.7
Alcohol or drug abuse (%)	3.9
**Medical history (%)**	
Heart failure	29.6
Myocardial infarction	8.1
Pacemaker	1.5
ICD	0.1
CRT-D	0.1
Dialysis	0.7
**Comorbidities (%)**	
**Cardiac**	
Coronary artery disease	60.6
Hypertension	53.8
Atrial fibrillation	35.9
Atrial flutter	1.4
Cardiac valvular disease	33.5
Pericardial diseases	2.7
**Non-cardiac**	
COPD or asthma	30.4
Dyslipidemia	50.4
Stroke/transient ischemic attack	20.1
Diabetes mellitus	19.9
Chronic renal insufficiency	16.5
Peripheral vascular disease	8.8
Cancer	3.6
Anemia	25.9

ICD indicates implantable cardioverter defibrillator; CRT-D, cardiac resynchronization therapy with defibrillator; COPD, chronic obstructive pulmonary disease; ACS, acute coronary syndrome.

© 2020 Gupta A et al. *JAMA Network Open.*

eTable 3. Patient-Level Adherence to Core Performance Measures for Inpatient Heart Failure Care

	**Eligible/contraindicated population** (n=10004)	**Overall** N,%
**Performance measures (%)**		
LVEF assessment	9840	6258 (63.6)
ACEI/ARB for LVSD at discharge	1266	648 (51.2)
β blocker for LVSD at discharge	1327	609 (45.9)
Evidence-based β blocker for LVSD at discharge	1327	262 (19.7)
Scheduled appointment at discharge	9662	2325 (24.1)

LVEF indicates left ventricular ejection fraction; ACEI, angiotensin-converting enzyme inhibitor; ARB, angiotensin receptor blocker; LVSD, left ventricular systolic disfunction;

© 2020 Gupta A et al. *JAMA Network Open.*

eFigure. Flowchart of Sampling, Data Collection, Cleaning, and Analysis

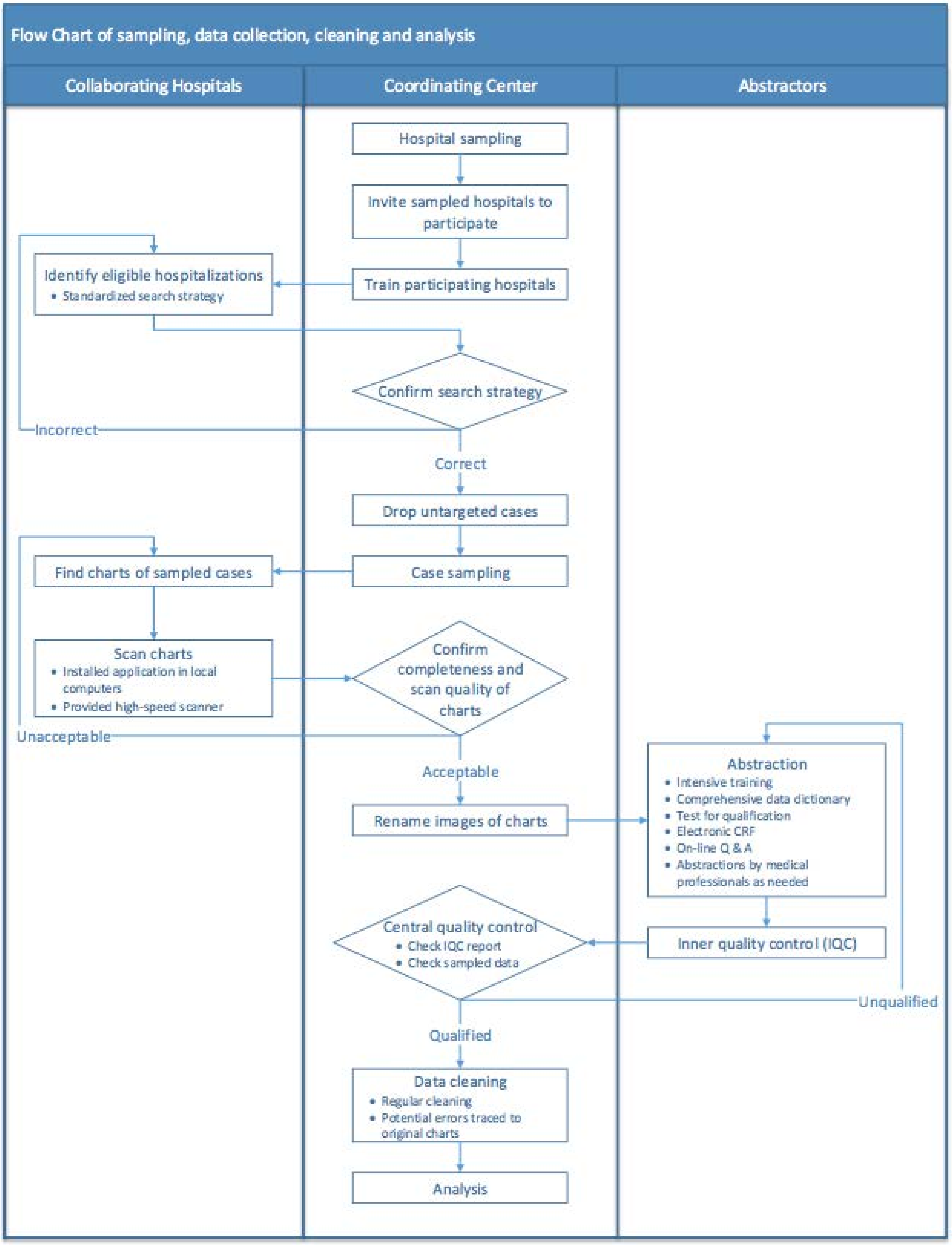

© 2020 Gupta A et al. *JAMA Network Open.*
